# Fabrication Process for Deep Submicron SQUID Circuits with Three Independent Niobium Layers

**DOI:** 10.3390/mi12040350

**Published:** 2021-03-24

**Authors:** Silke Wolter, Julian Linek, Josepha Altmann, Thomas Weimann, Sylke Bechstein, Reinhold Kleiner, Jörn Beyer, Dieter Koelle, Oliver Kieler

**Affiliations:** 1 Fachbereich Quantenelektronik, Physikalisch-Technische Bundesanstalt (PTB), 38116 Braunschweig, Germany; thomas.weimann@ptb.de (T.W.); oliver.kieler@ptb.de (O.K.); 2 Center for Quantum Science (CQ) and LISA+, Physikalisches Institut, Universität Tübingen, 72076 Tübingen, Germany; julian.linek@uni-tuebingen.de (J.L.); kleiner@uni-tuebingen.de (R.K.); koelle@uni-tuebingen.de (D.K.); 3 Fachbereich Kryosensorik, Physikalisch-Technische Bundesanstalt (PTB), 10587 Berlin, Germany; josepha.altmann@ptb.de (J.A.); sylke.bechstein@ptb.de (S.B.); joern.beyer@ptb.de (J.B.)

**Keywords:** low-temperature superconductors, nanotechnology, SQUID, niobium technology

## Abstract

We present a fabrication technology for nanoscale superconducting quantum interference devices (SQUIDs) with overdamped superconductor-normal metal-superconductor (SNS) trilayer Nb/HfTi/Nb Josephson junctions. A combination of electron-beam lithography with chemical-mechanical polishing and magnetron sputtering on thermally oxidized Si wafers is used to produce direct current SQUIDs with 100-nm-lateral dimensions for Nb lines and junctions. We extended the process from originally two to three independent Nb layers. This extension offers the possibility to realize superconducting vias to all Nb layers without the HfTi barrier, and hence to increase the density and complexity of circuit structures. We present results on the yield of this process and measurements of SQUID characteristics.

## 1. Introduction

Superconducting quantum interference devices (SQUIDs) are sensitive detectors of magnetic flux *Φ*, used in a large variety of applications [1,2]. Strongly miniaturized direct current (dc) SQUIDs with lateral size in the µm range (microSQUIDs) or even sub-µm range (nanoSQUIDs) have received increasing attention during the last years [3,4], as they offer high spatial resolution and high sensitivity for the detection and investigation of magnetic sources on the nanoscale. Accordingly, nanoSQUIDs have promising applications for high-resolution scanning SQUID microscopy [5,6,7,8,9,10,11,12,13] and for the detection and investigation of individual magnetic nanoparticles (MNPs), nanowires or nanotubes [14,15,16,17,18,19,20,21,22]. Due to their small SQUID loop and size of the Josephson junctions (JJs) intersecting the loop, nanoSQUIDs are suited for operation in strong external magnetic fields [23,24]. In addition, downscaling the linewidth of the SQUID loop provides improved coupling to MNPs [25,26,27] as the magnetic field of which decays cubically with the distance. For a magnetic dipole placed in 10 nm distance to the SQUID loop, nanoscale SQUIDs can provide spin sensitivities even below 1 µ_B_/Hz^1/2^ (µ_B_ is the Bohr magneton) [7].

Most micro- and nanoSQUIDs are based on single layer devices with constriction-type JJs (Dayem bridges). The use of sandwich-type trilayer JJs, typically with Nb electrodes, is less common. However, this approach allows one to use a mature multilayer technology, which offers the realization of more complex and advanced nanoSQUID layouts, such as three-axis vector nanoSQUIDs [28] and auxiliary components, such as gradiometric feedback loops and transformers [29] with high yield and reproducibility of JJ parameters. The challenge that comes with this approach is the need for the fabrication of deep submicron JJs, to enable operation in strong magnetic fields. Accordingly, one needs to achieve JJs with high critical current density *j*_0_, to provide large enough critical currents *I*_0_. During the last years, it has been shown that the above-mentioned requirements can be met by fabricating devices based on trilayer JJs with superconducting (S) Nb electrodes and a normal conducting (N) HfTi barrier, using nanopatterning by electron beam lithography (EBL) and chemical–mechanical polishing (CMP) [30,31]. The critical current density of SNS JJs with HfTi barriers can be adjusted over a very wide range by the thickness of the N layer up to *j*_0_ ≈ 1 MA/cm^2^ at temperature *T* = 4.2 K. Furthermore, lateral JJs sizes down to 80 nm diameter have been achieved [32,33]. We note that by the implementation of intrinsically shunted SNS JJs we avoid space-consuming external shunting resistors of the JJs in the circuit.

So far, our fabrication process has allowed for the realization of nanoSQUID designs with two independent Nb layers, i.e., Nb layers that are stacked on top of each other, separated by a highly insulating SiO_2_ layer, and that are patterned lithographically in separate steps. The two independent Nb layers can be connected vertically by SNS JJs with a HfTi barrier. For some applications, e.g., for the realization of nanoSQUID susceptometers, it is desirable to add at least one more independent Nb layer and to include vertical interconnections (vias) between the independent Nb layers, which are fully superconducting. There are already several Nb-multilayer processes which offer high yield for up to ten superconducting layers with sub-micron vias (see, e.g., Refs. [34,35,36,37]). Those processes use a combination of ultraviolet-lithography with multiple CMP steps to planarize dielectric interlayers. Our fabrication technology, which is based on the combination of EBL and CMP, by contrast, offers the possibility to realize even smaller vias and JJs. 

In this paper we describe the extension of our Nb multilayer technology for nanoSQUIDs from originally two to three independent layers of Nb. This allows to significantly increase the density of circuit structures. Additionally, the possibility to realize direct vias between all independent Nb layers, i.e. directly connecting each Nb layer without the normal conducting HfTi barrier in-between (e.g., to realize purely superconducting bridges for coils), offers improved design flexibility. We present results on the yield of our extended fabrication process and the possibility to realize passive on-chip components with high aspect ratio like interdigital capacitors (IDCs), which allow for the realization of capacitances matching the designed value with high accuracy. Additionally, we demonstrate that the extension of our fabrication process does not influence the electric transport properties of our SQUIDs and can therefore be used to further develop more complex nanoSQUID circuits.

## 2. Fabrication Technology

**Figure 1:**
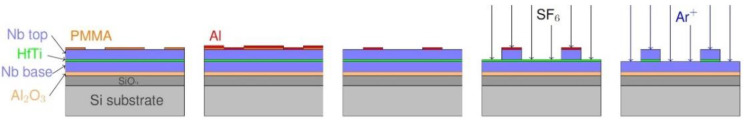
Layer scheme of the sub-µm range superconducting quantum interference device (nanoSQUID) fabrication process to define the Josephson junctions (JJs). After magnetron sputtering deposition (MSD) of the Al_2_O_3_ etch stop layer and the Nb-HfTi-Nb trilayer, the Al hard mask is patterned by a positive resist mask and a subsequent lift-off process and the Nb top layer of the trilayer is etched with inductively coupled plasma reactive ion etching (ICP RIE) using SF_6_. Subsequently, the HfTi barrier and the Al mask are removed by Ar ion beam etching (IBE).

**Figure 2:**
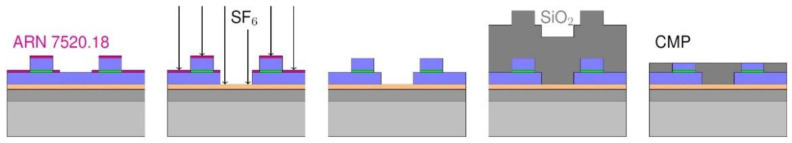
Layer scheme of the nanoSQUID fabrication process to pattern the Nb base layer and of the subsequent chemical–mechanical polishing (CMP) step. The Nb base layer is protected during the ICP RIE etching with SF_6_ by a high-resolution negative resist (ARN 7520.18) mask. Afterwards the mask is removed, and the wafer is covered with SiO_2_ via plasma enhanced chemical vapor deposition (PECVD). The wafer is planarized through the following CMP step.

**Figure 3:**
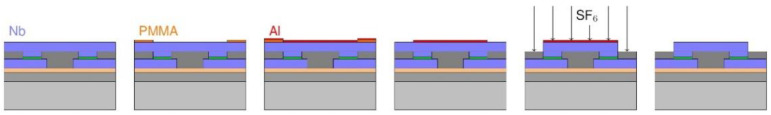
Layer scheme of the nanoSQUID fabrication process to define the first Nb wiring layer. After MSD of the 200 nm Nb layer, the Al hard mask is patterned by a positive resist mask and a subsequent lift-off process and the first Nb wiring layer is etched with ICP RIE using SF_6_. Subsequently, the Al mask is removed by using a wet etching step.

**Figure 4:**
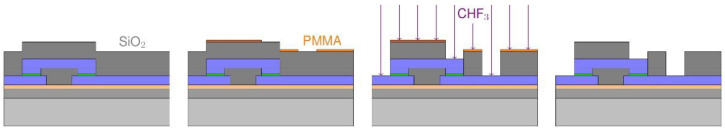
Layer scheme of the nanoSQUID fabrication process to etch windows (vias) into SiO_2_ to connect the Nb layers. SiO_2_ is deposed on top of the structure via PECVD and protected by a patterned positive resist mask during ICP RIE with CHF_3_.

**Figure 5:**
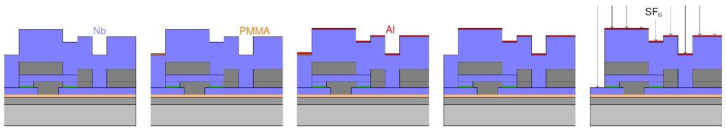
Layer scheme of the nanoSQUID fabrication process to define the second Nb wiring layer. After MSD of this third independent Nb layer the Al hard mask is patterned by a positive resist mask and a subsequent lift-off process and the second Nb wiring layer is etched with ICP RIE using SF_6_.

The fabrication technology here employs EBL to ensure high alignment precision for nanopatterning of multilayer Nb structures. In addition, a CMP step is used to planarize the SiO_2_ layer between the first and second independent Nb layer. Both technologies are available at the clean room center at PTB Braunschweig and have been applied before in the fabrication of JJ-based circuits [29,30,33]. Figure 1, Figure 2, Figure 3, Figure 4 and Figure 5 illustrate the deposition and patterning steps (we note that the layer thicknesses are not to scale).

We use 3-inch Si wafer substrates with 300 nm thermally oxidized SiO_2_ (Figure 1). After a pre-cleaning step of the substrate (in-situ Ar sputter cleaning: 50 W for 2 min), a 30 nm Al_2_O_3_ etch-stop layer is grown by magnetron sputtering deposition (MSD) in a high-vacuum chamber at room temperature. Subsequently the Nb-HfTi-Nb trilayer is grown by MSD. The trilayer consists of a 160 nm Nb base layer, 20 to 22 nm Hf_wt50%_Ti_wt50%_ (HfTi) and a 200 nm Nb top layer for six wafers, which were fabricated for this paper. As mentioned above, the HfTi thickness can be adjusted, to obtain a desired critical current density, in a typical range from 17 to 26 nm [33]. To define the deep sub-µm JJs, a 30 nm Al hard mask is patterned by lift-off, using EBL with PMMA as a positive resist. The Nb top layer is removed by inductively coupled plasma reactive ion etching (ICP RIE). To achieve optimum etching times, we use laser endpoint detection for ICP RIE of Nb and SiO_2_ layers. For etching the Nb top layer SF_6_ is used to obtain high etching rates and to ensure steep sidewalls approaching 90°. Ion beam etching (IBE) with Argon ions is applied to remove the HfTi barrier and simultaneously the Al hard mask.

To pattern the Nb base layer (Figure 2) it is crucial for our optimized fabrication process to use the high-resolution negative resist ARN 7520.18 (and optimized EBL doses and proximity correction) which allows the patterning of the desired small SQUID geometry. ICP RIE is applied and the Al_2_O_3_ layer below the Nb base acts as a reliable etch stop. After removing the negative resist mask (using BAKER REZI-38), the wafer is covered by about 600 nm SiO_2_ by plasma enhanced chemical vapor deposition (PECVD) to not only ensure isolation between the first and the second independent Nb layer, but also to serve as a planar underlay for the following layers. Therefore, CMP is used to planarize the SiO_2_ layer to the height of the Nb top layer. This way SiO_2_ is removed from the Nb top layer which can be contacted electrically by the upper Nb layers afterwards.

In the next step (Figure 3), MSD (with in-situ Ar sputter cleaning: 50 W for 1 min) is used to deposit the first 200 nm thick Nb wiring layer (Nb wiring 1) which is again patterned by a mask of positive resist to define another Al hard mask. Equivalent to the patterning of the Nb top layer, the first Nb wiring layer is etched by SF_6_ with ICP RIE. Subsequently the Al hard mask is removed by a wet etching process using the developer maD-332 resp. the acid-based etching solution TechniEtch Al80.

In the next fabrication step, we use PECVD again to deposit another 300 nm thick layer of SiO_2_ on top of the wafer (Figure 4) to ensure the isolation between the first and second Nb wiring layers. We use a positive resist mask patterned by EBL and etching by ICP RIE with CHF_3_ to create small windows (vias) in the SiO_2_ layer. Here we can open contact windows down to the Nb base layer and to the first Nb wiring layer.

As all Nb layers, Nb wiring 2 is deposited starting with an in-situ Ar sputter cleaning (50 W for 1 min) by MSD (Figure 5). The thickness of the second Nb wiring layer must be comparably large (800 nm) compared to the underlying layers to ensure that the superconducting Nb layer does not break off at the edges, because of the uneven structures when connecting the Nb base layer. For the last Nb etching step, another Al hard mask, which is again formed by an EBL patterned positive resist mask and a lift-off process, is needed. By ICP RIE with SF_6_ the second Nb wiring layer is patterned.

We note that an AuPd resistive layer can be integrated between the first and second Nb wiring layer fabrication step (not shown here) to form passive components such as resistors. By choosing the thickness of the AuPd layer, a desired sheet resistance can be adjusted; for example, a 70 nm AuPd layer results in a sheet resistance of 5 Ω.

In the following sections, we present measurement results concerning the yield and the functionality of parallel-plate capacitors to test the isolation between independent Nb layers and of vias to examine vertical connections between the three independent Nb layers. Furthermore, we investigated the realization of large fine pitch interdigital capacitors. Additionally, SQUIDs and single JJs were fabricated and investigated regarding their electrical transport characteristics. Six wafers were fabricated during the optimization of the presented process with slightly different process parameters like HfTi thicknesses and etching times.

## 3. Evaluation of Isolation and via Yield

**Figure 6:**
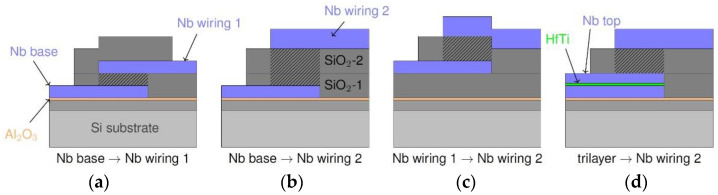
Layer scheme of the different types of parallel-plate capacitors. The plates of capacitors are in all three independent layers of Nb (**a**–**c**) as well as in the trilayer (**d**), and they are separated by SiO_2_ as the dielectric layer. The hatched area represents the volume between the plates.

In order to examine the quality of isolation and vias between Nb layers, we fabricated and characterized parallel-plate capacitor structures with varied dimensions, 10 µm × 10 µm to 800 µm × 800 µm, between all independent layers of Nb (base, wiring 1, wiring 2) and additionally between the trilayer and the second wiring layer. In Figure 6 the different types of capacitors are sketched.

First, we measured the resistance of the parallel-plate capacitors at room temperature with a wafer prober system (SÜSS MicroTec PM5 [38]) using a Keithley Model 2000 multimeter^1^. Thereby, we refer to the electrical contact between the three independent Nb layers as “open”, i.e., there are no unintentional shorts, if the measured resistance is larger than 120 MΩ. The results are summarized in Table 1. Furthermore, we note that we refer to a “high” yield even though it is less than 100%, since we fabricate devices for research purposes, not for industrial purposes, and the investigated less complex SQUIDs showed functionality (see Section 5). 

Those measurements showed a yield of 70% for a total of 443 investigated capacitors with different sizes and plates in Nb base and Nb wiring 1. For capacitors with plates in Nb base and Nb wiring 2, and for capacitors between the two wiring layers, a very high yield of 97% for 238 and 91% for 241 investigated capacitors was achieved, respectively. Capacitors with plates in the trilayer and Nb wiring 2 showed a high yield of 85% for a total of 47 investigated capacitors. The larger capacitors between the Nb base layer and the first wiring layer stemming from the middle of all wafers showed a slightly lower yield. We attribute this result to the inhomogeneity of our CMP step in which the center part of the wafer is somewhat stronger polished than the edges. This unevenness is likely to increase the occurrence of shorts through the insulation layer. Moreover, for some wafers the yield of capacitors between the two wiring layers was quite low. In those cases, clearly visible Niobium residuals (“fences”) appeared due to gaps between the first wiring layer and the dielectric and led to shorts between the wiring layers.

The capacitance *C* of selected parallel-plate capacitor structures from wafers 5 und 6 was measured at 4.2 K using a KEYSIGHT E4980A LCR-meter [38]. This was done to test the low-temperature functionality of the capacitors as well as to obtain an estimate of the thickness *d* of the two SiO_2_ layers (SiO_2_-1 and SiO_2_-2). To obtain *d*, the data were fitted according to

**Figure 7:**
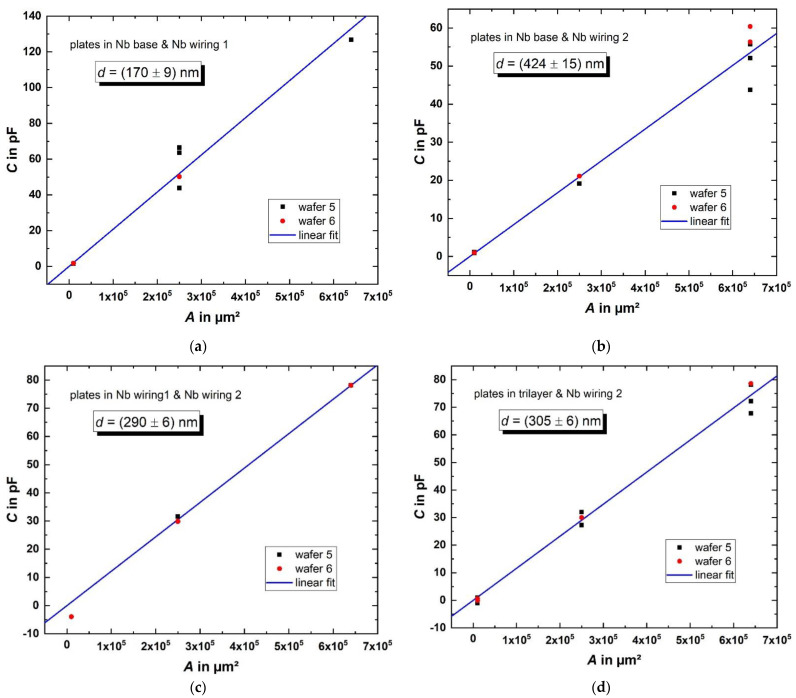
Measured capacitances (corrected values) of capacitors (corresponding to Figure 6) on wafers 5 and 6 with different sizes (100 µm × 100 µm … 800 µm × 800 µm). The thickness of the dielectric layers was calculated according to Equation (1) from the fit parameters for capacitors with plates in the Nb base and first wiring layer (**a**), in the Nb base and second wiring layer (**b**), in the two wiring layers (**c**) and in the trilayer and second wiring layer (**d**).

*C* = *ε*_r_*ε*_0_*A/d* + *offset* (1)

where *ε*_0_ is the dielectric constant of vacuum and *ε*_r_ = 4 [39] is the permittivity of our PECVD SiO_2_. Since *C*(*A* = 0) = 0 should be valid, we correct the measured capacitances by a constant offset, (33.6 ± 2.4) pF, which is assumed to be independent of the size of the capacitors and can be attributed to the parasitic capacitance of the measurement setup. We note that the offset partially results from so called “fringing fields” [40], whereas the effect is stronger for the smaller capacitors and therefore, the offset is strictly spoken not a constant value. However, since the deviation though the effect of the fringing fields for our capacitors is not notable (only about 1.3% of the capacitance for the smallest capacitors) compared to the measurement error, we treat the offset like a constant here. The corrected values and the linear fit functions (Equation (1) for offset = 0) are shown in Figure 7 for all four types of capacitors as a function of the area *A* of the parallel plates of the capacitors.

The effective SiO_2_ thickness between the different capacitors with plates in the Nb base layer and the first Nb wiring layer (Figure 7a) is *d* = (170 ± 9) nm. For capacitors with plates in the Nb base and second wiring layer (Figure 7b), an effective thickness of *d* = (424 ± 15) nm SiO_2_ between the two plates is estimated. Since the SiO_2_-2 layer has a nominal thickness of 300 nm, there is a significant mismatch between the estimated thickness of the SiO_2_-1 layer from the measurement shown in Figure 7a and the estimation of the thickness of the SiO_2_-1 layer from the measurement shown in Figure 7b, reduced by the nominal thickness of the SiO_2_-2 layer. The inter-wafer inhomogeneity of the CMP process causes the thickness of the SiO_2_-1 layer of wafer 5 and wafer 6 to be less predictable. Additionally, the intra-wafer inhomogeneity of the CMP process depends on the structure dimensions and density, causing the thickness of the remaining SiO_2_-1 layer to differ across each wafer. Both effects can be observed in Figure 7b, where the capacitances are larger for wafer 6 than for wafer 5 due to stronger polishing, and the spread of the measured capacitances is stronger for larger structures.

For capacitors with plates in both wiring layers (Figure 7c) and for capacitors with plates in the trilayer and the second wiring layer (Figure 7d), we obtain *d* = (290 ± 6) and (305 ± 6) nm, which is 97% and 101%, respectively, of the intended thickness of the SiO_2_-2 layer. The differences to the desired value are within the accuracy of the measurement. Since no CMP step was used for those capacitors, the thickness of the deposited SiO_2_-2 layer could be adjusted precisely and therefore, the capacitances match the designed values accurately.

Altogether, the fabricated capacitor structures show no drastic nonlinear dependence of the capacitance on the area of the parallel plates. The thickness of the dielectric SiO_2_-2 layer could be estimated within the scope of measurement accuracy and matches the expectations. The estimation of the thickness of the SiO_2_-1 layer shows a larger uncertainty due to the inhomogeneity of the CMP process, but not the area of the capacitors’ plates which can be fabricated with a very high accuracy.

**Figure 8:**
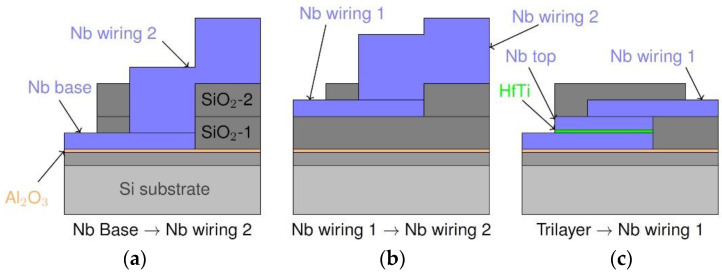
Layer scheme of the different types of vias connecting three independent layers of Nb. The connections from the second Nb wiring layer to the Nb base (**a**) and first Nb wiring layer (**b**) are fully superconducting; the connection between Nb wiring 1 and Nb base (**c**) is disrupted by a normal conducting HfTi barrier.

In addition to examining the quality of the isolation between the Nb layers, we studied the yield characteristics of direct vias connecting the three independent layers of Nb as well as of JJs of varied size. The direct connections from Nb wiring 2 to Nb wiring 1 and to the Nb base layer avoid contact through the normal conducting barrier layer (Figure 8a,b). These superconducting vias were designed to vary in size from 1 µm × 1 µm to 10 µm × 10 µm. The JJs, or "JJ vias" (contacts opened by CMP), with lateral dimensions 0.1 µm × 0.1 µm to 5 µm × 5 µm, naturally include the HfTi barrier (Figure 8c).

To investigate the yield of functional vias (Nb vias, connecting Nb wiring 2 with Nb base or Nb wiring 1, and JJ vias, connecting Nb wiring 1 with Nb base), we measured the resistance of the vias including their on-chip leads at room temperature with a wafer prober system. The expected resistance is *R* ≈ 90 Ω, which corresponds to the resistance of the on-chip leads, calculated from the sheet resistance of niobium. The HfTi barrier is expected to result in an additional resistance of about 20 Ω. Therefore, we define a via as “functional” if the measured resistance does not exceed the expected total resistance of the on-chip leads and the HfTi barrier by more than 25% (i.e., *R* ≈ 140 Ω). The total number and the yield of functional vias is presented in Table 2.

A yield of 88% for a total of 242 vias in different sizes connecting the Nb base layer and the second wiring layer was achieved. Vias connecting the two wiring layers got a high yield of 95% for a total of 240 measured vias with different sizes. The investigation of the JJ vias showed a yield of 38% for 128 structures.

To assess the functional quality of the Nb vias also at low temperatures, transport current tests were performed at 4.2 K on one exemplary Nb via, connecting the Nb base layer and Nb wiring 2, with lateral size of 2 µm × 2 µm from wafer 2. A bias current up to 100 mA, corresponding to a current density of up to 2.5 × 10^6^ A/cm^2^, could be applied without a measurable voltage drop. This demonstrates the expected significant increase in critical current density for direct Nb vias compared to JJ vias. We note that all vias (superconductor-superconductor and superconductor-normal metal-superconductor vias), which were functional at room temperature and which were investigated at low temperature (including vias which are parts of devices) showed superconducting properties.

As discussed in Section 2, there is only one SiO_2_ plasma etching step within the fabrication process (see Figure 4). Note, that for contacting from the Nb wiring 2 layer to the Nb wiring 1 layer, only the SiO_2_-2 layer must be removed whereas for contacting to the Nb base layer, the SiO_2_-2 layer as well as the SiO_2_-1 layer must be etched. As a consequence, an appropriate etching time need to be applied to open windows both to the Nb base layer as well as to the Nb wiring 1 layer. Furthermore, the thickness of the SiO_2_-1 layer is inhomogeneous across the wafers due to CMP. This situation makes it difficult to find an optimal etching time. Hence, we observe a smaller yield for Nb base to Nb wiring 2 connections for some wafers, where the etching time was not sufficient to open all vias down to the Nb base layer. For the vias connecting the two wiring layers, a yield of 95% was determined. However, since we observed so-called Nb fences here, the apparently high yield could be caused to a certain extent by unintentional shorts between the Nb layers. The yield of JJ vias is high for the first two wafers, but a higher inhomogeneity of CMP for the wafers 3 to 6 lead to a significant decrease in functional JJ vias.

In summary, the overall yield of parallel-plate capacitors and Nb vias is very high (83% for all capacitors and 91% for all Nb vias) and might be increased in the future by optimizing etching times or, e.g., by introducing an additional CMP step. In general, the laser endpoint detection for etching SiO_2_ and Nb is extremely important for the fabrication process. However, the optimum etching time is difficult to find due to inhomogeneous layer thicknesses and etching rates.

## 4. Characterization of Structures with High Aspect Ratio

The process described above was also used to realize fine pitch IDCs in the Nb base layer with extremely high aspect ratio *AR* = *n* × *L*/*p*. Here, *n* × *L* is the total length of the IDC, i.e., the total number of fingers on both sides of the IDC (*n*) times the length *L* of one finger. The pitch *p* is defined as the distance between the centers of two neighboring fingers, i.e., *p* is the sum of the linewidth of one finger and the gap between two fingers. We fabricated IDCs with *L* = 4183 µm, *n* = 500, 750, and 1000 and with *p* = 1.4, 2, and 4 µm for different IDCs with the same *n*.

**Figure 9:**
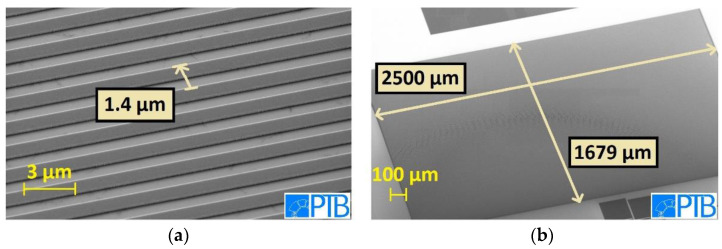
Scanning electron microscopy (SEM) images of a fine pitch interdigital capacitors (IDC) fabricated in the Nb base layer. (**a**) The IDC’s pitch = 1.4 µm equals the design value. (**b**) The large IDC shown here exhibits no visible defects.

An AR of up to 2.2 × 10^6^ (with *n* = 750 and *p* = 1.4 µm) was achieved. Figure 9 shows scanning electron microscopy (SEM) images of a fabricated IDC, indicating that large structures with small linewidths can be fabricated without any visible defects. The capacitance of the IDCs was measured in liquid He at *T* = 4.2 K with an LCR-meter. To estimate *C* from the geometry of the IDCs, we used the relation ([41] p. 96, Equation (4.47a)):

*C*[pF] *=* 3.937 · 10^−5^ · *L*(*ε_r_* + 1) · {0.11(*n −* 3) + 0.252}     for *L* in µm, (2)

with *n* = {500, 750, 1000} and *L* = 4183 µm, which is valid for IDCs with linewidth = gap such as the IDCs presented here. For the effective dielectric constant of the structures, *ε_r_* ≈ 10 was estimated in advance for the layout, as the value of the Si-substrate *ε_r,Si_* ≈ 12 is slightly reduced by the smaller value *ε_r,SiO_*_2_ ≈ 4 [39] of the insulating SiO_2_ on top of the IDCs and between the fingers.

**Figure 10:**
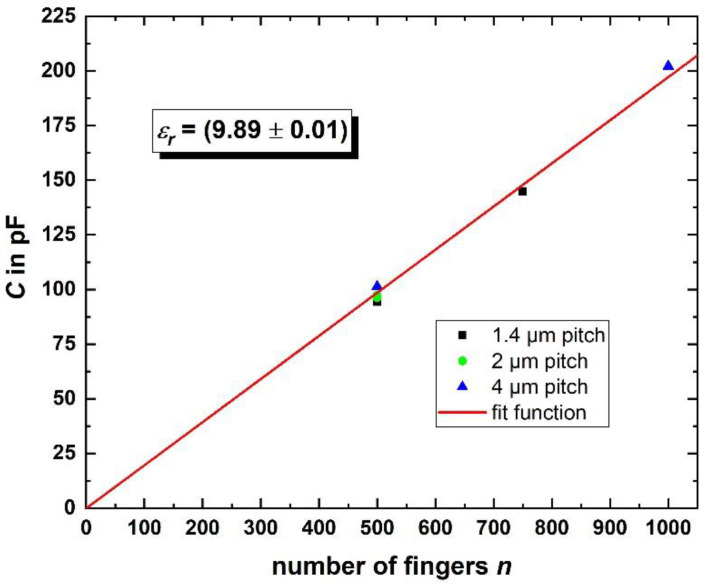
Measured capacitance of the investigated IDCs with different pitches. With a linear fit function according to Equation (2), the effective dielectric constant of the capacitors’ dielectric can be estimated.

The measured capacitances, reduced by an offset (33.6 pF), which is attributed to the parasitic capacitances of the measuring system, are shown in Figure 10. We used a linear fit function according to Equation (2) to derive the effective dielectric constant *ε_r,eff_* = (9.89 ± 0.01), which deviates only 1% from the value, which was estimated for the layout beforehand. Regarding the capacitances, for IDCs with *n* = 500, we expected 100 pF per design. The mean value of the measured capacitances for these IDCs, having different pitches (1.4 µm, 2 µm, and 4 µm), was (97 pF ± 3) pF.

Furthermore, we define an IDC as “functional”, if there are no unintentional shorts, i.e., the measured resistance is larger than 120 MΩ. The yield of functional IDCs was found to be ≈ 14%. We attribute this comparably low yield to the large area of the structure that is vulnerable to small particles masking the Nb between the fingers during the etching step. Nevertheless, the results demonstrate functional IDCs with capacitance values matching the designed values with high accuracy.

## 5. Determination of Basic Electric Transport Properties of SQUID Test Structures Based on Submicron Josephson Junctions

To demonstrate the suitability of the presented fabrication process for the realization of nanoSQUIDs we designed [42], fabricated, and characterized dc SQUID gradiometers with SNS JJs with nominal lateral JJ size of 200 nm × 200 nm. A closed superconducting loop is constructed in the Nb base layer and is connected via two JJs to the modulation line in the first Nb wiring layer. This results in a parallel gradiometer. The SQUID inductance *L_SQ_* is then given by one half of the inductance of one loop of the gradiometer. The magnetic flux *Φ* in the SQUID can be adjusted by the modulation current *I_mod_* through the modulation line. Since the Nb wiring 2 layer is not needed for this SQUID design, the SQUIDs are covered by the SiO_2_-2 layer. We show that the extension of our process from originally two to three independent layers of Nb does not downgrade the electric transport properties of our fabricated devices.

**Figure 11:**
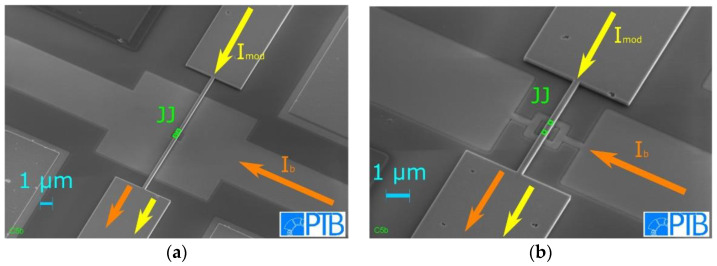
SEM images of both types of fabricated dc SQUID parallel gradiometers, (**a**) SQ-1 and (**b**) SQ-2 after the fabrication of the Nb wiring 1 layer. A closed superconducting loop in the Nb base layer is connected via two JJs to a modulation line in the Nb wiring 1 layer. The bias current *I_b_*, and the modulation current *I_mod_* are indicated by arrows and the positions of the JJs are indicated by squares. The voltage across the SQUID is measured between the bias current terminals.

The SQUID loop of the first type of SQUID gradiometer (SQ-1 in Figure 11a) was designed to be 10 µm x 10 µm large and to have a linewidth of 4.6 µm. A second type of SQUID gradiometer (SQ-2 in Figure 11b) was fabricated with a much smaller 1.5 µm × 1.5 µm outer loop size with significantly reduced linewidth of 250 nm. In addition to the SQUIDs, single SNS JJs with the same nominal area of 0.04 µm^2^ were fabricated to investigate their electric transport characteristics. All data shown below were taken at liquid He temperature (*T* = 4.2 K).

**Figure 12:**
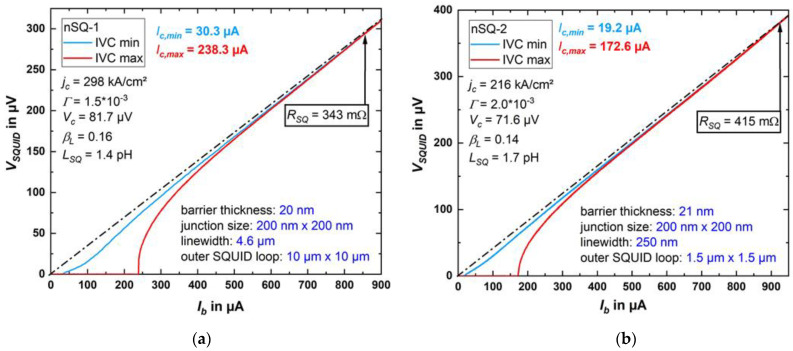
The current–voltage characteristics (IVCs) at 4.2 K of SQ-1 (**a**) and SQ-2 (**b**), with *I_mod_* adjusted to achieve maximum (IVC max) and minimum (IVC min) critical current. The characteristic electric transport and geometric parameters of the SQUIDs are indicated.

The current–voltage characteristics (IVCs), with *I_mod_* adjusted to obtain maximum critical current *I_c,max_* and minimum critical current *I_c,min_*, are shown in Figure 12a for an exemplary SQUID of the type SQ-1 with JJs having a barrier thickness of *d*_HfTi_ = 20 nm and in Figure 12b for another exemplary SQUID (type SQ-2) with a slightly thicker HfTi barrier (*d*_HfTi_ = 21 nm). The IVCs with maximum critical current can be well described within the resistively and capacitively shunted junction (RCSJ) model [43,44], with negligible capacitance and with negligible noise rounding, which is consistent with the small noise parameter *Γ* = 2*πk_B_T*/( *I_0_*Φ_0_) ≈ 2 × 10^−3^ (with the Boltzmann constant *k_B_* and average single JJ critical current *I_0_* = *I_c,max_*/2). The junctions exhibit a critical current density *j_c_* ≈ 298 kA/cm^2^ and *j_c_* ≈ 216 kA/cm^2^ for SQ-1 and SQ-2, respectively. They have a normal resistance *R_SQ_* ≈ 343 mΩ (SQ-1) up to 415 mΩ (SQ-2), leading to characteristic voltages in the range of *V_c_* = *I_c,max_R_SQ_* ≈ 82 µV (SQ-1) resp. 72 µV (SQ-2).

**Figure 13:**
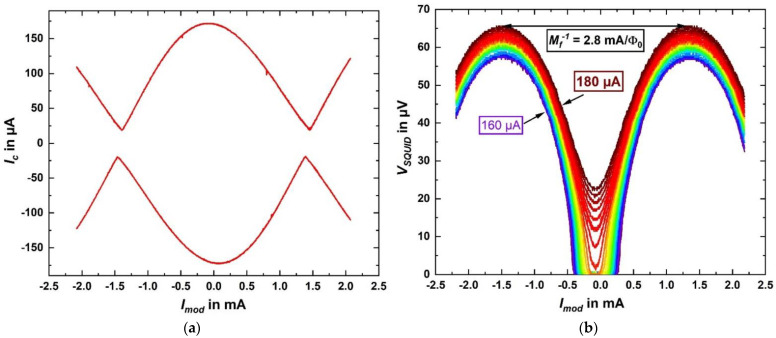
SQUID oscillations of SQ-2 at 4.2 K. (**a**) Critical current vs. modulation current. (**b**) Voltage vs. modulation current for constant bias currents (from 160 to 180 µA, in 1 µA steps).

For SQ-2, we show in Figure 13a the critical current oscillations *I_c_(I_mod_)*; Figure 13b shows voltage oscillations *V(I_mod_)* for different values of fixed bias current close to *I_c,max_*. From the oscillation period (current *I_mod,0_* required to couple one flux quantum Φ_0_ = h/2e ≈ 2.0678 × 10^−15^ Vs to the SQUID) we determine the inverse mutual inductance M_f_^−1^ = *I_mod,0_*/Φ_0_ between the modulation line and the SQUID, which ranges from about 2 mA/Φ_0_ (SQ-1) to 2.8 mA/Φ_0_ (SQ-2) for the two exemplary SQUIDs. We note that M_f_^−1^ for nSQ-1 is larger than for SQ-2, which may be due to flux focusing, caused by the larger washer of SQ-1 [45]. For SQ-2, the small shift Δ*I_mod_* = ±83 µA of the maxima in *I_c_*(*I_mod_*) for opposite polarity (c.f. Figure 13a) is, as expected, very close to the symmetric single JJ critical current *I_c,max_*/2 = 86.3 µA. This means that the asymmetry in the *I_c_*(*I_mod_*) oscillations is likely dominated by inductance asymmetry due to asymmetric current biasing of the SQUIDs, and that an asymmetry in the critical currents of the two JJs is negligible.

For negligible noise rounding and negligible critical current asymmetry, we can then estimate the screening parameter

*β_L_* = *I_c,max_L_SQ_*/Φ_0_. (3)

*β_L_* is derived using the relation between the normalized modulation depth Δ*I_c_*/*I_c,max_* vs. *β_L_* (with Δ*I_c_* = *I_c,max_* − *I_c,min_*), which has been obtained from numerical simulations based on the RCSJ model [1]. From Equation (3), we then obtain *L_SQ_* ≈ 1.4 pH (SQ-1) resp. 1.7 pH (SQ-2). The maximum modulation voltage of our exemplary SQUIDs is 71 µV for SQ-1 and 61 µV for SQ-2 (c.f. Figure 13b) and the derived maximum transfer coefficients are *V_Φ_* = 944 µV/Φ_0_ (SQ-1) and 441 µV/Φ_0_ (SQ-2). All characteristic parameters for both SQUIDs are summarized in Table 3 together with results from ref. [42], where nominally the same layout was fabricated, but with a two-Nb-layer fabrication process. The deviations from our results (regarding the mutual inductance, for example) can be explained, since the SQUIDs were fabricated with another deposition system using nominally an identical HfTi target. However, in general, the SQUID characteristics of the SQUIDs which were investigated for this paper are in accordance with the parameters from ref. [42].

The critical current densities of both investigated SQUIDs stemming from different wafers meet the expectations regarding data from former measurements of critical current densities of single JJs [32,33] (fabricated with the process for only two independent Nb layers), including different wafers with varying thickness of the HfTi barrier of the JJs. In addition to 16 SQUIDs of the types SQ-1 and SQ-2 from wafer 1 and wafer 2, where we demonstrated a high yield for JJ vias, a total of eight single JJs from the same two wafers with a HfTi barrier thickness of 20 nm or 21 nm were investigated. For 10 SQUIDs with *d_HfTi_* = 20 nm, the critical current density was *j_c_* = (309 ± 57) kA/cm^2^ and *j_c_* = (243 ± 46) kA/cm^2^ for 6 SQUIDs with *d_HfTi_* = 21 nm. The critical current density of five single JJs was (261 ± 41) kA/cm^2^ for the thinner barrier and (170 ± 10) kA/cm^2^ for three JJs with the thicker barrier.

**Figure 14:**
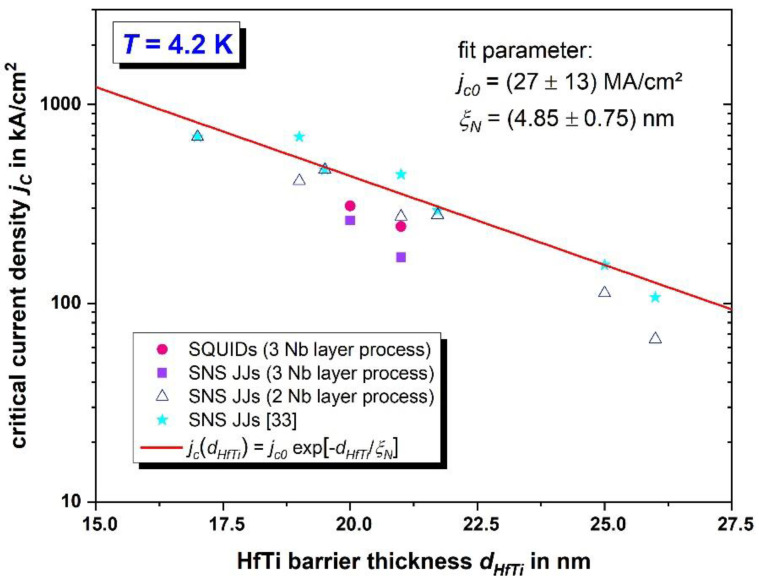
Critical current densities at 4.2 K vs. HfTi barrier thickness of SQUID types SQ-1 and SQ-2 and single HfTi SNS-JJs, all with nominal JJ width of 200 nm.

The mean value of *j*_c_(*d*_HfTi_) for all SQUIDs and for single JJs from wafer 1 and wafer 2 measured here and the critical current density of single JJs with the same nominal width, but fabricated with the process for two independent Nb layers and with different HfTi barrier thicknesses, are shown in Figure 14. The mean values of the SQUIDs and single JJs, which were prepared with the extended fabrication process, agree reasonably well with the data of the single JJs, which were fabricated with the two Nb layer process.

Additionally, data of single JJs with nominal widths of 200 nm from [33] are shown for comparison. For those, *j*_c_ is calculated using the effective area (considering deviations from the nominal area due to the patterning process and due to edge damage).

For our JJs, the expression [46]

*j_c_* = *j_c0_* · exp[−*d_HfTi_/ξ_N_*] (4)

can be used [33] to describe the dependence of the critical current density on the HfTi barrier thickness, where *ξ*_N_ is the coherence length in the normal conducting barrier. In ref. [33], the fitting parameters are *j_c0_* = (27 ± 13) MA/cm^2^ and *ξ_N_* = (4.85 ± 0.75) nm. In general, the fit function agrees well with our measurement results for SQUIDs and single JJs. Since our data were determined using the designed area, which is somewhat larger than the effective area of the JJs, our data are slightly below the curve from ref. [33].

## 6. Summary and Outlook

We extended our fabrication technology for nanoSQUIDs with SNS JJs from originally two to three independent layers of Nb. Thereby, six test wafers were fabricated to optimize the process and to assess the high yield and characteristics of capacitor structures and vias as well as single Josephson junctions and dc SQUIDs.

The circuits on all six wafers showed a high yield of investigated parallel-plate capacitors having different sizes and plates in all possible combinations of Nb layers. Measurements of the capacitance of those capacitors were performed to deduce the height of the dielectric SiO_2_ layer between the capacitors’ plates. The deviation from of the designed values can be explained by the inhomogeneity of the CMP process, causing a difference in height of SiO_2_ across the wafers. Fabricating high aspect ratio IDCs was challenging due to the large area of the structures which must be fabricated without any defects, causing a reduced yield; still we demonstrate the possibility to obtain the capacitances very precisely in agreement with the capacitor design. Furthermore, we studied the yield of Nb vias and JJ vias connecting the three independent layers of Nb, which was very high for all investigated Nb vias. For JJ vias, which were fabricated using a CMP process, enabling additionally much smaller dimensions, the yield suffered much more from inter-wafer inhomogeneity (polishing grade).

To demonstrate the suitability of our new fabrication process for the realization of SQUIDs, we fabricated dc SQUID gradiometer within three fabrication runs for two wafers, respectively, and investigated the JJ and SQUID characteristics. Since all investigated SQUIDs showed reasonable critical currents and modulation upon applying a modulation current, we conclude a very high yield for the first fabrication run. The two subsequent fabrication runs showed an exceedingly lower yield due to a too strong or too weak polishing, indicating that the CMP fabrication step is very crucial to achieve a high yield for nanoSQUIDs. Still, the deviation of the critical current density from the designed values for all investigated SQUIDs and single JJs from the first fabrication run was small. Therefore, we conclude that the performance of SQUIDs and single JJs is not affected by the extension of the fabrication process from originally two to three independent layers of Nb, since they show IVCs which fully match our expectations regarding their HfTi barrier thicknesses. In addition, the suitability of our fabrication process for different types of precise on-chip capacitors, which can be used as auxiliary components for more complex SQUID circuits, could be demonstrated.

The Nb multilayer technology presented in this paper offers the possibility to increase the density of structures on one wafer to create more complex and advanced nanoSQUID circuits, e.g., three-dimensional vector nanoSQUIDs [28] and nanoSQUID susceptometers [29].

In future work we expect to improve upon the yield by enhancing the controllability of the CMP process by introducing additional supporting structures as well as by adapting the etching times to optimize the steepness of the structures’ sidewalls. Furthermore, we will optimize the design of the dc SQUID gradiometers to increase the coupling to magnetic nanoparticles, and we will perform detailed studies of nanoSQUID noise properties.

## Figures and Tables

**Table 1:** Total number of investigated capacitors between different Nb layers for 6 different wafers and yield for each type of capacitor (corresponding to Figure 6) without shorts.

Capacitors	Wafer 1	Wafer 2	Wafer 3	Wafer 4	Wafer 5	Wafer 6
**(a) Base** **→ Wiring 1**	205	200	10	10	9	9
	(**71%**)	(**73%**)	(**50%**)	(**30%**)	(**67%**)	(**56%**)
**(b) Base** **→ Wiring 2**	100	100	10	10	9	9
	(**100%**)	(**97%**)	(**90%**)	(**80%**)	(**89%**)	(**100%**)
**(c) Wiring 1** **→ Wiring 2**	100	103	10	10	9	9
	(**100%**)	(**100%**)	(**70%**)	(**0%**)	(**22%**)	(**78%**)
**(d) Trilayer** **→ Wiring 2**	9	--	10	10	9	9
	(**100%**)		(**80%**)	(**80%**)	(**100%**)	(**67%**)

**Table 2:** Total number of investigated vias for 6 different wafers (corresponding to Figure 8) and yield for each type of via.

Capacitors	Wafer 1	Wafer 2	Wafer 3	Wafer 4	Wafer 5	Wafer 6
**(a) Base** **→ Wiring 2**	104	100	10	10	9	9
	(**93%**)	(**80%**)	(**80%**)	(**100%**)	(**100%**)	(**89%**)
**(b) Wiring 1** **→ Wiring 2**	102	100	10	10	9	9
	(**95%**)	(**99%**)	(**100%**)	(**100%**)	(**100%**)	(**44%**)
**(c) Trilayer** **→ Wiring 1 (JJ vias)**	21	21	22	22	21	21
	(**90%**)	(**81%**)	(**18%**)	(**18%**)	(**10%**)	(**10%**)

**Table 3:** Characteristic parameters of two representative SQUIDs and a SQUID from ref. [42] with the same layout for comparison.

	*d_HfTi_* (nm)	*I_c,min_* (µA)	*I_c,max_* (µA)	*j_c_* (kA/cm^2^)	*R_SQ_* (mΩ)	*V*_c_ (µV)	*V_Φ_* (µV/Φ_0_)	*1*/*M*_f_ (mA/Φ_0_)	*β_L_*	*L*_SQ_ (pH)
**SQ-1**	20	30.3	238.3	298	343	81.7	944	2.0	0.16	1.4
**SQ-2**	21	19.2	172.6	216	415	71.6	441	2.8	0.14	1.7
**ref. [42]**	24	21	178	233	233	41.5	100	4.4	0.18	2.1

## Data Availability

The data presented in this study are available on request from the corresponding author. The data are not publicly available because there is no public cloud memory available at the federal institute of PTB.
